# Privacy-Protection Scheme of a Credit-Investigation System Based on Blockchain

**DOI:** 10.3390/e23121657

**Published:** 2021-12-09

**Authors:** Ke Yuan, Yingjie Yan, Tong Xiao, Wenchao Zhang, Sufang Zhou, Chunfu Jia

**Affiliations:** 1 School of Computer and Information Engineering, Henan University, Kaifeng 475004, China; yuanke@henu.edu.cn (K.Y.); yanyingjie@henu.edu.cn (Y.Y.); xiaotong@henu.edu.cn (T.X.); zhangwenchao@henu.edu.cn (W.Z.); 2 Henan Key Laboratory of Big Data Analysis and Processing, Henan University, Kaifeng 475004, China; 3 College of Cybersecurity, Nankai University, Tianjin 300350, China; cfjia@nankai.edu.cn

**Keywords:** blockchain, smart contract, zero-knowledge proof, searchable-symmetric encryption

## Abstract

In response to the rapid growth of credit-investigation data, data redundancy among credit-investigation agencies, privacy leakages of credit-investigation data subjects, and data security risks have been reported. This study proposes a privacy-protection scheme for a credit-investigation system based on blockchain technology, which realizes the secure sharing of credit-investigation data among multiple entities such as credit-investigation users, credit-investigation agencies, and cloud service providers. This scheme is based on blockchain technology to solve the problem of islanding of credit-investigation data and is based on zero-knowledge-proof technology, which works by submitting a proof to the smart contract to achieve anonymous identity authentication, ensuring that the identity privacy of credit-investigation users is not disclosed; this scheme is also based on searchable-symmetric-encryption technology to realize the retrieval of the ciphertext of the credit-investigation data. A security analysis showed that this scheme guarantees the confidentiality, the availability, the tamper-proofability, and the ciphertext searchability of credit-investigation data, as well as the fairness and anonymity of identity authentication in the credit-investigation data query. An efficiency analysis showed that, compared with similar identity-authentication schemes, the proof key of this scheme is smaller, and the verification time is shorter. Compared with similar ciphertext-retrieval schemes, the time for this scheme to generate indexes and trapdoors and return search results is significantly shorter.

## 1. Introduction

The credit system is one of the symbols that constructs economic and social progress and development, and credit has gradually become everyone’s second ID card [1]. Credit investigation reflects a person’s credit status, and it has stepped into all aspects of our life. A credit-investigation agency provides convenient credit-inquiry services to users by legally collecting and processing the credit information of users from the credit investigation system.

With the development of Internet finance, we have entered the era of data, and credit data have shown explosive growth. The traditional credit-investigation system takes a few credit-investigation agencies as the main body, and its stored credit-investigation information is seriously unable to meet the needs of users. There is an island phenomenon among credit-investigation systems, and the format of their credit-investigation information is also different. These conditions make it impossible to share credit-investigation information. In addition, the traditional credit-investigation system relies on a centralized server, which means that once it is attacked by hackers, the entire system will fall down and cannot work. Credit-investigation information is sensitive and private, so the credit-investigation system needs security and privacy protection.

Blockchain and smart contracts can solve the problem of information silos and the risks brought by the centralized server in the traditional credit-investigation system. In the current credit-investigation system, financial institutions provide credit-investigation user inquiries and related credit services. Credit-investigation agencies act as credit-investigation data providers, and they encrypt the data and upload them to the cloud. However, this multi-entity system will lead to complex interactions, which is not conducive to the sharing of credit-investigation information. Blockchain technology has the characteristics of traceability, privacy protection, and avoidance of single points of failure as a secure, distributed ledger based on cryptography. A smart contract is programmable code that cannot be tampered with running on the blockchain, which greatly expands the application fields of the blockchain. Smart contracts are deeply integrated with credit investigation, and they help to realize the safe sharing of credit data and to reduce the cost of credit-data collection.

The identity authentication of the traditional credit-investigation system will lead to a certain risk of identity information leakage. For example, the method of entering the account and password for identity authentication and login in the credit-investigation system will cause a user’s identity information to be intercepted by the adversary, thereby jeopardizing the security of the credit-investigation system. The method of identity authentication through biometrics such as face and fingerprint has the advantages of not being forgotten or lost and is easy to use anytime and anywhere. However, because the biological characteristics remain unchanged for many years and accompany each individual throughout his life, its security cannot be guaranteed when there are loopholes or when the system database is attacked [2]. Although the blockchain provides pseudonyms that have nothing to do with the information of credit-investigation users, it cannot fully realize the privacy of users’ identity. Therefore, the current credit-investigation system has an urgent need to solve the security problem of identity authentication.

The data in the credit-investigation system are sensitive, so malicious cloud service providers will spy on and tamper with user’s data. To protect the privacy of the data, we need to encrypt them before uploading them to the cloud servers. Although the ciphertext can ensure the security of credit-investigation data, it consumes a significant amount of bandwidth resources when the ciphertext is searched. Therefore, we need to search the ciphertext and return the search results to the user, while ensuring the privacy and security of the data to the greatest extent.

Zero-knowledge-proof technology [3] can ensure the security and privacy of credit-investigation users in the identity-authentication phase, and it can protect their identities from eavesdropping and acquisition by malicious adversaries. zkSNARKs [4], as a zero-knowledge-proof application tool, allows credit users to prove to the system that the “thesis” is correct by describing a particular “thesis,” but the judgment will not reveal any valid information. Users submit their own identity information by using zero-knowledge-proof technology; then, the smart contract will verify the user’s identity. Once the verification is passed, the user will be anonymously authenticated, and the blockchain will record that the user’s address is legal.

Searchable-symmetric-encryption technology can improve the search efficiency of cloud service providers and can realize data searches in ciphertext data. In addition, this technology guarantees data security and prevents attacks from malicious cloud service providers and other adversaries.

This scheme combines blockchain, zero-knowledge-proof, and searchable-symmetric-encryption technology to develop a securer credit information-sharing scheme.

### 1.1. Related Works

Blockchain is a horizontal and connected technology, which can promote interconnection among various industries and fields and the development of other technologies [5,6]. Xu et al. [7] proposed a social-credit-investigation system based on blockchain technology, which enabled smart contracts for identity verification and authorization. Li et al. [8] proposed a reputation blockchain ecosystem to implement autonomous credit and established a credit-evaluation model. Zhang et al. [9] designed a personal-credit-investigation-information-sharing-platform framework based on blockchain 3.0 architecture and implemented a credit-blacklist-sharing mechanism. Zhu [10] proposed an identity-authentication and intelligent-credit-reporting method based on blockchain technology, which realized multi-dimensional-identity-security authentication and a distributed ledger of credit ratings.

Faisca and Rogado [11] proposed an end-to-end-identity-authentication mechanism based on the JSON web token and blockchain technology. The token can use “claims” to encode personal-cloud- and customer-related information in a secure way. Cui et al. [12] proposed a blockchain-based multi-WSN authentication scheme for the Internet of Things to achieve mutual authentication of node identities in various communication scenarios. Abbasi and Khan [13] proposed a VeidBlock1 scheme, which generates verifiable identities by following a reliable authentication process. All identities created by VeidBlock are verifiable and anonymous, so this scheme protects the user’s privacy during the verification and authentication phases. Zhang et al. [14] stored users’ identities on the blockchain and stored the encrypted personal information outside the blockchain; however, the user’s identity information was directly exposed on the blockchain, and malicious attacks can easily steal the user’s identity. Zhou et al. [15] proposed an improved key-distribution solution (blockchain with identity-based encryption (BIBE)). BIBE separates the nodes in the chain to complete a user’s identity verification and private-key protection. Mikula [16] proposed an identity and access-management system using blockchain technology to support the identity verification and authorization of entities in the digital system. Gabay et al. [17] proposed a scheme based on blockchain technology and smart contracts, and they achieved privacy protection authentication through a zero-knowledge=proof method based on tokens and the Pederson commitment. Wan et al. [18] proposed zk-DASNARK and realized the data-feedback-feed scheme of zero-knowledge proof based on zk-DASNARK to ensure the privacy and authenticity of smart contracts.

Li et al. [19] proposed a blind-signature scheme suitable for a blockchain system against quantum attacks, and this scheme improved the security and privacy of the blockchain. Li et al. [20] proposed a searchable-symmetric-encryption scheme based on blockchain technology, which not only improved the efficiency of data retrieval but also ensured the fairness of both parties’ transactions. Gao et al. [21] proposed an attribute-based encryption scheme based on blockchain technology to achieve trusted-access control of data while ensuring an access strategy and attribute privacy. Agyekum et al. [22] proposed a proxy re-encryption method to ensure data-sharing security in a cloud environment.

### 1.2. Contribution

The goal of this study was to provide a privacy-protection scheme for a credit-investigation system based on blockchain technology to ensure the secure sharing of data between the credit-investigation user and the credit-investigation agency. The main contributions are summarized as follows.

This study proposed a privacy-protection scheme for the credit-investigation system based on blockchain technology. The information-silo problem caused by the centralized-server method was solved by using the decentralization and non-tamperable characteristics of the blockchain, and the credit-investigation data was protected from being tampered with by malicious cloud service providers.

This scheme adopted identity-authentication technology based on zero-knowledge proof—credit-investigation users can prove that they are legal users without revealing any private identity information. This scheme also used a searchable-symmetric-encryption technology, which ensures the secure storage and the efficient searching of credit-investigation data.

Compared with some existing schemes, this scheme achieved better privacy protection. Compared with zk-DASNARK, the cost of this scheme in the identity-authentication phase is affordable. The cost of this scheme in the searchable-encryption process is significantly lower than other schemes.

## 2. Basic Knowledge

### 2.1. Blockchain and Smart Contracts

Blockchain, as the underlying technology of Bitcoin [23], is a distributed ledger based on cryptography security with convenient validation and tamper-free features. Blockchain can also be considered as a list sorted by some sort of consensus, which is accessible to any node. A smart contract [24] is a program running on the blockchain network and is a collection of code and data (status). Through the realization of calculation and storage through smart contracts, a large number of applications on the Ethereum blockchain have become a reality. As a segment of code that can be uploaded and executed, it has tamper-free and fully distributed properties, and developers efficiently develop blockchain-related applications by deploying smart contracts.

### 2.2. Zero-Knowledge Proof

Zero-knowledge proof [3] implies that the prover can convince the verifier that a certain statement is correct through interaction with the verifier, but the verifier knows nothing about the statement otherwise. Zero-knowledge proof has three characteristics: completeness, soundness, and a state of zero knowledge.

(1) Completeness. If the prover does have the answer to a certain argument, he can definitely find a method to prove to the verifier that the data he holds are correct.

(2) Soundness. If the prover does not have the answer to a certain conclusion at all, he cannot (or can only with a very low probability) convince the verifier that the purported answer in his hands is correct.

(3) Zero knowledge. The verifier only knows that a certain assertion is correct or wrong, but he knows nothing about the assertion.

### 2.3. zkSNARKs

The acronym zkSNARKs [4] stands for zero-knowledge succinct non-interactive argument of knowledge, which is a form of zero-knowledge proof that is more concise and applicable in non-interactive environments. This logic was first proposed in 2012, and zkSNARKs can be implemented in the blockchain environment. In the case of blockchain transactions using zkSNARKs, the validity of the transaction can be transmitted to nodes other than the sending and receiving nodes without exposing information such as the receiver, the sender, and the transfer amount. The content of zkSNARKs is mainly divided into two parts: one part is to convert the problem to be proved into a circuit, and the other part is to convert the circuit into a verifiable mathematical polynomial problem.

### 2.4. Searchable-Symmetric Encryption

The traditional searchable-symmetric-encryption algorithm [25] has three participant entities: the data owner, the server, and the user. The data owner has a document set $D=(D_{1},D_{2},\cdots ,D_{n})$, and the cipher text set $C=(C_{1},C_{2},\cdots ,C_{n})$ is generated by the key *K*. In addition, an encrypted index *I* is also generated. Then, he sends *C*, *I* to the server. The data owner and the user share the key *K*. When the user wants to search for a document containing the keyword *W*, the key *K* is used to generate a search trapdoor $t_{i}$ for the keyword *W*, and the trapdoor is sent to the server. The server uses a search algorithm to search for ciphertext *C* through $t_{i}$ and *I*. Finally, the user decrypts locally to obtain the plaintext *D*.

## 3. System Model

The privacy-protection scheme of the blockchain-based credit investigation system includes five entities including credit-investigation agencies, credit-investigation users, the blockchain, cloud service providers, and financial institutions.

Regarding the credit-investigation agency, we assumed that the credit-investigation agencies (CIA) are completely trustworthy. The credit-investigation agencies (CIA) have a large number of user-credit-reporting reports and can safely own, control, and conditionally provide credit reporting of users’ personal-credit information, in addition to obtaining relevant fees in the process.

The credit-investigation user ($u_{i}$) is a typical data consumer. He needs to pass identity authentication and entrust financial institutions to inquire about the relevant credit-investigation information.

The smart contract in the blockchain (BC) can verify the identity of the credit-investigation users, and the blockchain network connects to other physical nodes. The blockchain stores the hash digest of the ciphertext of the credit-investigation user’s information to ensure that the data can be traced and that they cannot be tampered with.

We assumed that the cloud service provider (CSP) is not trustworthy. It stores the ciphertext of the user’s credit-investigation information and returns the ciphertext that meets the requirements based on the user’s trapdoor information. In addition, it receives credit-investigation information but may dishonestly perform the tasks assigned in the system.

Financial institutions include commercial banks, etc. We assumed that financial institutions (FI) are completely trusted. In this scheme, financial institutions provide credit-investigation information inquiry and credit services to credit-investigation users, but they cannot authorize other credit-investigation users to query services.

**Definition** **1.** *This scheme is composed of the algorithm eight-tuple (KeyGen,Enc,Setup, Prove,Authenticate,Trapdoor,Search,and Dec), part of which is based on a searchable- symmetric-encryption algorithm [26]. The formal description is as follows:*

*$KeyGen(1^{k})\to K$. The algorithm is a probabilistic key-generation algorithm. It takes a security parameter k as input and outputs a key array K.*

*$Enc\left(K,D_{i}\right)\to \left(I,c_{w_{i}}\right)$. The algorithm is an encryption algorithm. It takes a plaintext $D_{i}$ and a key K as input and outputs the index I and the ciphertext $c_{w_{i}}$.*

*$Setup\left(1^{\lambda },C\right)\to \left(EK_{i},VK_{i}\right)$. This algorithm is a key-generation algorithm of zkSNARKs. It takes a security parameter λ and a circuit C as input and outputs a proof key $EK_{i}$ and a verification key $VK_{i}$.*

*$Prove(EK_{i},id_{i},sign\left(id_{i}\right),Timestamp)\to \pi$. This algorithm is a proof algorithm of zkSNARKs. It takes a proof key $EK_{i}$, a user’s identity information $id_{i}$, identity-information signature $sign(id_{i})$, and timestamp Timestamp as input and outputs a proof π.*

*$Authenticate\left(VK_{i},\pi \right)\to \left(true/false\right)$. This algorithm is a verification algorithm of zkSNARKs. It takes as input a verification key $VK_{i}$ and a proof π to verify whether the verification is successful.*

*$Trpdoor\left(K,w_{i}\right)\to t_{i}$. This algorithm is a trapdoor-generation algorithm executed by the financial institution FI that accepts the credit-investigation user’s entrustment. It takes a key K and the search keyword $w_{i}$, and it outputs the trapdoor $t_{i}$.*

*$Search\left(I,t_{i}\right)\to c_{w_{i}}$. The algorithm is a ciphertext search algorithm executed by the cloud service provider CSP that accepts the credit-investigation user’s entrustment. It takes a search index I and a trapdoor $t_{i}$ as input, and it outputs the ciphertext $c_{w_{i}}$ that meets the requirements.*

*$Dec\left(K,c_{w_{i}}\right)\to D_{i}$. The algorithm is a decryption algorithm executed by the financial institution FI that accepts the credit-investigation user’s entrustment. It takes the secret key K and ciphertext $c_{w_{i}}$ as input and outputs the plaintext $D_{i}$.*

This scheme is based on blockchain technology, smart-contract technology [27], searchable-symmetric-encryption technology, and zkSNARKs to realize the identity authentication of credit-investigation users and the secure sharing of credit data in the credit-investigation system.

**Figure 1:**
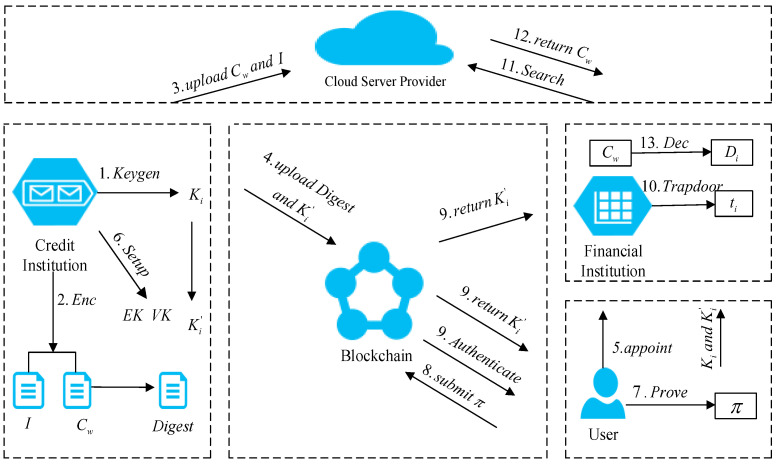
System model.

To facilitate understanding, in our plan, we instantiated a blockchain-based credit-investigation-system plan, as shown in Figure 1. Credit-investigation agencies CIA have a large number of credit-investigation users’ credit investigation information $D=(D_{1},D_{2},\cdots ,D_{n})$.

In the data-encryption stage, the credit-investigation agency first generates key *K* according to algorithm Keygen and generates ciphertext $c_{w_{i}}$ and index *I* through algorithm Enc, which are stored by the cloud service provider CSP. Then, the key *K* is encrypted according to the public key of the credit-investigation user to obtain $K^{{}^{\prime }}$, and the ciphertext $c_{w_{i}}$ is hashed to obtain the hash value $h_{w_{i}}$ before being saved on the blockchain.

At the registration stage, the credit-investigation user $u_{i}$ needs to register with the credit-investigation agency in advance. In addition, credit-investigation users need to entrust financial institutions to perform data-query services.

In the zero-knowledge key-generation stage, the credit-investigation agency must design and develop a circuit that conforms to the user’s identity, and it must generate a proof key $EK_{i}$ and a verification key $VK_{i}$ through the Setup algorithm. Finally, the proof key is distributed to credit-investigation users, and the verification key is distributed to the verification contract.

In the proof-generation stage, when the credit-investigation user $u_{i}$ passes the identity authentication without revealing his specific identity information, he needs to enter his identity information in the Prove algorithm to generate a zero-knowledge proof π conforming to the circuit, and he needs to submit it to the verification contract.

In the identity-authentication phase, the verification contract verifies that π and the proof key are correct through the Authenticate algorithm, and the ciphertext $K^{{}^{\prime }}$ is sent to the credit-investigation user and the financial institution; additionally, the identity of the credit-investigation user $u_{i}$ is legal. Otherwise, the identity authentication cannot be passed.

In acquiring the trapdoor phase, the credit-investigation user receives $K^{{}^{\prime }}$, decrypts it to obtain the key *K*, and then returns to the financial institution through the secure channel. Financial institutions use the Trapdoor algorithm to generate trapdoor $t_{i}$ and send it to cloud service providers.

At the stage of obtaining the ciphertext, the cloud service provider uses the Search algorithm to return the corresponding ciphertext $c_{w_{i}}$ to the financial institution.

In the decryption stage, the financial institution decrypts the ciphertext through the Enc algorithm to obtain the credit-investigation information $D_{i}$, returns it to the relevant credit investigation user $u_{i}$, and provides relevant credit services.

## 4. Detail Scheme

To facilitate the understanding of these notations, the notations used in this article are shown in Table 1.

### 4.1. System Initialization

Set *p* to be a large prime number and $G_{p}$ to be the only subgroup of $Z_{p}^{*}$, and then select generator $g\in G_{p}$, random number $r\in Z_{p}$, security parameter *k* and hash function $H:{\left\{0,1\right\}}^{*}\to Z_{p}$. Suppose the public key of the credit-investigation user $u_{i}$ is $PK_{i}$, and the private key is $SK_{i}$.

Credit-investigation users, financial institutions, and credit-investigation agencies participate in the blockchain and obtain the blockchain address. These entities act as nodes to jointly build an alliance blockchain together. All nodes that maintain the blockchain network can upload data to the blockchain only through the practical-byzantine-fault-tolerance (PBFT) consensus mechanism.

### 4.2. Data Encryption

The credit-investigation agency CIA selects the security parameter *K* and uses the $KeyGen(1^{k})\to K$ algorithm to randomly generate the key $K=(K_{1,i},K_{2,i},K_{3,i},K_{4,i})$ for credit-investigation user $u_{i}$.

We chose a pseudo-random function *f* and two pseudo-random permutations φ and ϕ. We scanned the credit data set $D=\{D_{1},...,D_{n}\}$ and generated the keyword set δ(D) of the document. For each different keyword $w_{i}\in \delta \left(D\right)$, we generated the corresponding dictionary sequence table $D(w_{i})$ and set the global counter ctr=1.

CIA has a large number of credit-investigation data sets $D=\{D_{1},...,D_{n}\}$ and uses the $Enc(K_{4,i},D_{i})$ algorithm to generate ciphertext $c_{w_{i}}$ and index *I*, where 1≤i≤n. The Enc algorithm uses the AES symmetric-encryption algorithm. Index *I* is composed of array *A* and lookup table *T*.

#### 4.2.1. Create an Array

Array A contains a linked list $L_{i}$ including node $N_{i,j}$, set $id(D_{i,j})$ to be the *j*th identifier of $D(w_{i})$ and generates key $K_{i,j}\leftarrow KeyGen\left(1^{k}\right)$. Node $N_{i,j}=\langle id\left(D_{i,j}\right)\left|\right|K_{i,j}\left|\right|\varphi_{K_{1}}\left(ctr+1\right)\rangle$ was created, where $1\leq j\leq \left|D(w_{i})\right|-1$. Node $N_{i,j}$ was encrypted to get $A\left[\varphi_{K_{1}}\left(ctr\right)\right]=Enc_{K_{i,j}}\left(N_{i,j}\right)$. Then, the last node $N_{i,j}=\langle id\left(D_{i,\left|D(w_{i})\right|}\right)\left|\right|0^{k}||NULL\rangle$ was created, and the node was encrypted to get $A\left[\varphi_{K_{1}}\left(ctr\right)\right]=Enc_{K_{i,\left|D(w_{i})\right|-1}}\left(N_{i,\left|D(w_{i})\right|}\right)$. Finally, all the nodes $N_{i,j}$ of the linked list $L_{i}$ were stored in the array A in a random order.

#### 4.2.2. Create a Query Table

We generated the query table $T\left[\pi_{K_{3}}\left(w_{i}\right)\right]=\langle addr_{A}\left(N_{i,1}\right)\left|\right|K_{i,1}\rangle ⊕f_{K_{2}}$ with the keyword $w_{i}\in \delta \left(D\right)$. Among them, the query table was composed of a two-tuple <address,value>, value presents the position $addr_{A}\left(N_{i,1}\right)$ of the array *A* and the decryption key $K_{i,1}$ of the node in $L_{i}$, and <address,value> presents the address $f_{K_{2}}\left(w_{i}\right)$ of *T*.

The credit-investigation agency CIA uploads the ciphertext $c_{w_{i}}$ and index *I* to the cloud service provider CSP, and it uploads the ciphertext digest $h_{w_{i}}$ of $c_{w_{i}}$, the ciphertext $K_{i}^{{}^{\prime }}$, and the ciphertext of the searchable-symmetric-encryption key to the blockchain.

$$h_{w_{i}}=Hash\left(c_{w_{i}}\right)$$

### 4.3. Registration

We assumed that the user also needs to register with the credit-investigation agency CIA in advance. Normally, a valid voucher or identification is required. Once the credit-investigation user $u_{i}$ passes the identity authentication, as a legitimate user, he will not repeatedly submit his own specific identity information during the scheduling or decryption stage, and he will remain anonymous. After registration, the credit-investigation user $u_{i}$ sends an entrusted service request and related keyword $w_{i}$ to the financial institution.

### 4.4. Zero-Knowledge Key Generation

**Figure 2:**
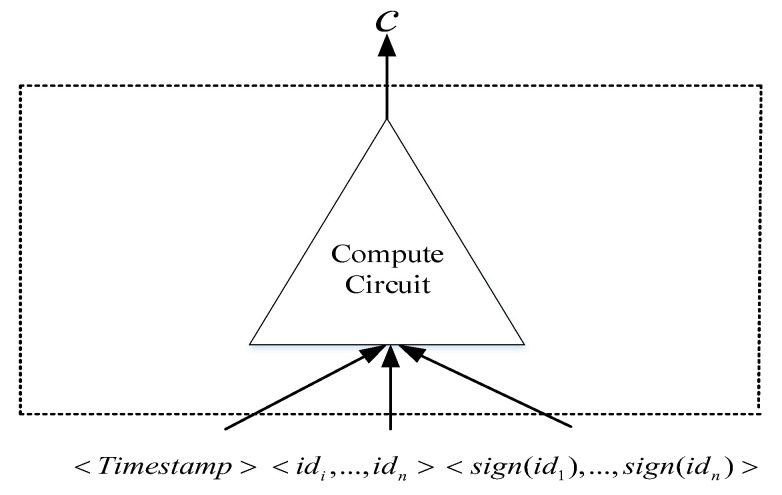
Compute circuit.

After the credit-investigation user completes the registration with the credit-investigation agency CIA, CIA needs to pre-design and develop a domain-specific language (DSL)-program that meets the zero-knowledge proof to generate circuit $C_{1}$ and $C_{2}$. Then, the circuit $C_{1}$ is composed of a calculation circuit, and the circuit $C_{2}$ is composed of a calculation circuit and a Sha256 circuit. In the circuit $C_{1}$, the credit-investigation user’s identity information set $<id_{1},\cdots ,id_{n}>$, identity information signature set $<sign\left(id_{1}\right),\cdots ,sign\left(id_{n}\right)>$, and the timestamp <Timestamp> are input into the compute circuit for calculation; then, the calculated value *h* is output to verify the authenticity and availability of the data. The structure of circuit $C_{1}$ is shown in Figure 2.

**Figure 3:**
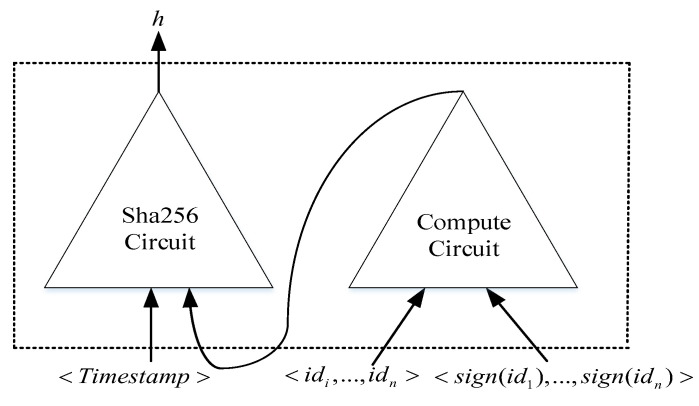
Sha256 circuit.

In the circuit $C_{2}$, the credit-investigation user’s identity information set $<id_{1},\cdots ,id_{n}>$ and information-signature set $<sign\left(id_{1}\right),\cdots ,sign\left(id_{n}\right)>$ are input to the compute circuit; both the timestamp <Timestamp> and the results obtained from the compute circuit are input into the Sha256 circuit and output the calculated hash value *h*. This value verifies the authenticity and availability of the identity-information set, the corresponding identity-information-signature set, and the timestamp of the credit-investigation user. The structure of circuit $C_{2}$ is shown in Figure 3.

Take the acquired security parameter λ and one of the $C_{1}$ and $C_{2}$ as input, then the proof key $EK_{i}$ and the verification key $VK_{i}$ are output. The credit-investigation agency CIA sends $EK_{i}$ to the credit-investigation user, then it creates a verification contract and sends $VK_{i}$ to the verification contract. This verification contract is public on the blockchain network and is used to verify whether the identity of the credit-investigation user $u_{i}$ is legal or not.

### 4.5. Generate the Proof

There are two types of input: public input and private input. The personal-identity information $id_{i}$ and $sign(id_{i})$ of the credit-investigation user $u_{i}$ are referred to as private input, and the timestamp is referred to as public input to prevent potential replay attacks and man-in-the-middle attacks. $u_{i}$ must input the correct $id_{i}$, $sign(id_{i})$, Timestamp, and $EK_{i}$ to generate a credible zero-knowledge proof π. This process is performed outside the blockchain and will not be written into the blockchain.

### 4.6. Identity Authentication

The credit-investigation user $u_{i}$ submits the zero-knowledge proof π to the verification contract for anonymous identity authentication. The smart contract verifies the zero-knowledge proof π and the verification key $VK_{i}$ without the participation of a third party. If the user-identity authentication is correct, the smart contract sends $u_{i}$’s key ciphertext $K_{i}^{{}^{\prime }}$ to $u_{i}$ and the financial institution, then it determines that the credit-investigation user $u_{i}$’s identity is legal, otherwise it will record that the user is illegal and cannot proceed to the next-step operation. The record of all identity authentication performed by the smart contract is stored on the blockchain, and the process will only reveal the address information of the user $u_{i}$ but will not reveal any identity information about $u_{i}$.

### 4.7. Obtain the Trapdoor

The credit-investigation user $u_{i}$ uses his private key $SK_{i}$ to decrypt $K_{i}^{{}^{\prime }}$ to obtain $K_{4,i}$ and then sends $K_{i}^{{}^{\prime }}$ and $K_{4,i}$ to the financial institution through a secure channel. Then, the $K_{i}^{{}^{\prime }}$ sent by $u_{i}$ has a time limit to ensure that he is the credit-investigation user authenticated by the verification contract just now. If the financial institution succeeds in the verification, then a trapdoor $t_{i}$ is generated according to the key $K_{4,i}$ and the corresponding keyword $w_{i}$. Finally, the financial institution submits $t_{i}$ and credit-investigation-information-inquiry fees to the cloud service provider.

### 4.8. Obtain the Ciphertext

After paying the inquiry fee, the cloud service provider receives the trapdoor $t_{i}$ sent by the financial institution FI, retrieves the corresponding ciphertext $c_{w_{i}}$ through the Search algorithm, and finally returns the ciphertext to FI.

In order to verify that the cloud service provider CSP has not tampered with the integrity and availability of the credit investigation ciphertext data and ciphertext data in the transmission process, the ciphertext $c_{w_{i}}$ needs to be hashed to obtain $Hash(c_{w_{i}})$. The result obtained by $Hash(c_{w_{i}})$ with the ciphertext digest $h_{w_{i}}$ in the blockchain is compared. If $h_{w_{i}}==Hash\left(c_{w_{i}}\right)$, it proves that the cloud service provider CSP has not tampered with the ciphertext data.

### 4.9. Decrypt the ciphertext

The financial institution decrypts the ciphertext with the key $K_{4,i}$ to obtain the credit-investigation information $D_{i}$ and sends it to the user $u_{i}$ through a secure channel. In addition, the financial institution provides related credit services to $u_{i}$ according to the credit-investigation report.

## 5. Performance Analysis

In this section, we perform a performance analysis of this scheme in two aspects.

(1) Since the cloud service providers set up in this scheme are malicious and dishonest, they will analyze and speculate or even tamper with the data of credit-investigation users. Malicious nodes in the blockchain will steal the data of users and impersonate other users. In view of the above situation, we should analyze whether the scheme meets the security and privacy-protection requirements based on the blockchain credit-investigation system.

(2) We first compared the characteristics of the credit-investigation system with the scheme [9] and the scheme [10], and then we analyzed the efficiency of the identity-authentication phase and compared it with zk-DASNARK [18]. Finally, we compared the cost of the searchable encryption phase with similar ciphertext-searchable schemes [20].

### 5.1. Security Analysis

**Theorem** **1.** *This scheme can realize the confidentiality of credit-investigation data.*

**Proof.**  Credit-investigation data are all searchable and symmetrically encrypted. Only credit-investigation users $u_{i}$ with successful authentication can obtain the key $K_{4,i}$. Other users cannot get the key, and even if they have keywords $w_{i}$ and trapdoor-generation algorithms, they cannot generate a trapdoor $t_{i}$. Moreover, $t_{i}$ is encrypted so that a malicious cloud service provider cannot decrypt or infer the credit-investigation ciphertext from the index without the key. The theorem is proved. □

**Theorem** **2.** *This scheme can realize the availability of credit-investigation data.*

**Proof.**  In this scheme, only successfully authenticated entities can obtain credit-investigation information. Specifically, credit-investigation users $u_{i}$ can generate a fully credible zero-knowledge proof π based on their identity information and can submit it to the verification contract of the blockchain. If the identity verified $u_{i}$ by the verification contract is legal, $u_{i}$ can obtain the key. Finally, $u_{i}$ entrusts financial institutions to send a trapdoor to cloud service providers to obtain and decrypt ciphertext $c_{w_{i}}$. Therefore, this scheme guarantees the availability of credit-investigation data. The theorem is proved. □

**Theorem** **3.** *This scheme can realize the tamper-proofability and the traceability of credit-investigation data.*

**Proof.**  The Merkle tree of the blockchain ensures the tamper-proofability of credit-investigation ciphertext. When a malicious cloud service provider CSP wants to tamper with credit-investigation ciphertext, the ciphertext cannot correspond to the ciphertext summary stored in the blockchain. Once the credit-investigation data are recorded by the blockchain, this information can be queried and traced. The theorem is proved. □

**Theorem** **4.** *This scheme can realize the ciphertext retrievability of credit-investigation data.*

**Proof.**  In this scheme, the financial institution needs to obtain authorization from the authenticated credit user and then obtain the relevant key and generate the relevant trapdoor. The cloud service provider retrieves the credit-investigation ciphertext. The theorem is proved. □

**Theorem** **5.** *This scheme can realize the fairness and ciphertext retrievability between credit-investigation users and cloud service providers.*

In traditional searchable encryption schemes, it is assumed that the cloud server will honestly perform the retrieval task and return the corresponding results. However, the cloud server may be malicious, meaning it does not return the search result or it return the wrong search result after receiving the retrieval task submitted by the user, causing the user to not receive the corresponding service.

**Proof.**  In this scheme, credit-investigation users can only obtain the ciphertext from the cloud service providers CSP by successfully delivering the credit-investigation fee. When CSP performs a retrieval task and returns search results to financial institutions, if the returned result is incorrect or the corresponding result is empty, then the behavior of CSP maliciously returning wrong results or blanks will be recorded on the blockchain. Therefore, CSP will return the correct retrieval results. In addition, financial institutions can retrieve the corresponding credit ciphertext through the trapdoor. The theorem is proved. □

**Theorem** **6.** *This scheme can realize the anonymity and authentication of identity in a credit-investigation data query.*

**Proof.**  Credit-investigation user $u_{i}$ conducts anonymous identity authentication through zero-knowledge-proof technology, and other irrelevant nodes only know that the user’s identity is legal. This scheme uses timestamps Timestamp and $id_{i}$ signatures as input to prevent potential replay attacks. The theorem is proved. □

### 5.2. Efficiency Analysis

This scheme was systematically compared with the blockchain-based credit-investigation system and traditional credit investigation, as shown in Table 2. Compared with the blockchain-based credit-investigation system [9], this scheme can achieve identity anonymity and verifiability, as well as ciphertext retrievability. Compared with the blockchain-based credit investigation system [10], this scheme also enables ciphertext retrievability and identity anonymity.

We used a computer with an Intel Core i7-8750H CPU @ 2.2 GHz with 16 GB memory for efficiency analysis. Zokrates [28] is an all-in-one tool to apply zkSNARKs to the blockchain. We used Zokrates and the smart contract on the Ethereum test network Rinkeby for identity-authentication experiments.

We analyzed the efficiency of 8 inputs and 16 inputs on circuit A and 16 inputs on circuit B. We mainly analyzed the number of constraints, and the time consumed for compilation in the zero-knowledge-proof phase, key generation, proof generation, and authentication, as shown in Table 3. In addition, we also analyzed the size of the proof key, the verification key, and the size of the proof, as shown in Table 4. We repeated each operation 50 times and took the average.

It can be seen from Table 3 that the verification time was 0.006 s. Even if the number of constraints keeps increasing, the time consumed by verification does not increase, which greatly improves the scalability of the scheme. The time consumed by other phases increases with the number of constraints. Moreover, the number of constraints is related to the circuits generated by zkSNARKs, and circuit $C_{2}$ is more complex than circuit $C_{1}$. The greater the number of constraints, the higher the security of identity authentication. In addition, we used the identity information $id_{i}$ and its signature as private input, and the timestamp Timestamp as public input to prevent potential replay attacks.

It can be seen from Table 4 that the size of the proof key, the verification key, and the proof increased with the number of inputs. Specifically, the maximum verification key was 4.0 KB, the maximum proof key was 6.4 MB, and the maximum space occupied by the proof was no more than 1.9 KB, which were all in an acceptable range.

This scheme was compared with zk-DASNARK [18], as shown in Table 5. Since both proof schemes are based on Sha256 circuits, we considered using the circuit $C_{2}$. The space occupied by this scheme to generate the verification key was almost equal to that of zk-DASNARK, while the time consumed to generate the proof was not much different from that of zk-DASNARK. Although this scheme consumed about twice as much time as zk-DASNARK in the key generation phase, zk-DASNARK consumed six times as much time as this scheme in the verification phase. Moreover, the key size generated by zk-DASNARK was eight times larger than that of this scheme.

**Figure 4:**
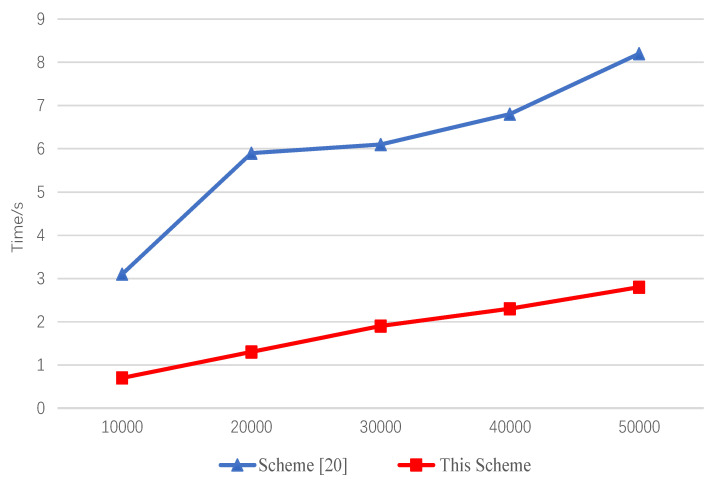
Time of build of an index for keywords with different size.

We implemented a searchable-symmetric-encryption process using Python 3.8 and compared it with another searchable-symmetric scheme based on blockchain technology [20]. We repeated each operation 50 times and took the average time. Figure 4 shows the time to generate indexes for both schemes, with keywords ranging from 10,000 to 50,000. With the increase in the number of keywords, the index time generated by the two schemes also increased, and the time of this scheme was generally shorter than that of scheme [20].

**Figure 5:**
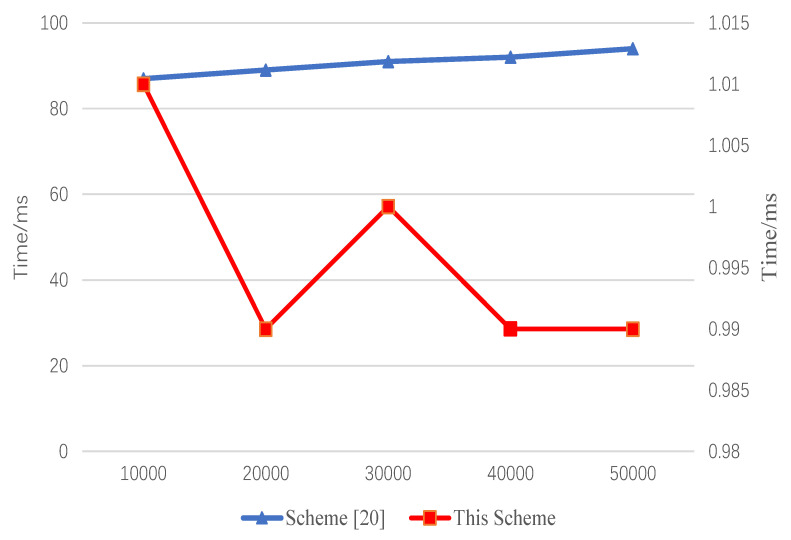
Time of build of a trapdoor and return search results for keywords with different sizes.

We analyzed the efficiency of the process of generating trapdoors and returning search results. The keywords ranged from 10,000 to 50,000. As shown in Figure 5, the time consumed by this scheme was basically about 1 ms. Because the scheme [20] needs to generate search transactions through a smart contract, the time consumed by this scheme to generate trapdoors and return search results was significantly less than that of scheme [20].

We took the compile operation entered by 16-Sha256 as the basic unit and calculated the approximate ratio of other operations to the compile operation. Since there are five different input numbers in the searchable-symmetric-encryption process, we set the number of keywords to 30,000. The specific data are shown in Table 6.

## 6. Conclusions

This study proposed a security and privacy-protection scheme for the credit-investigation system based on blockchain technology by combining technologies such as zero-knowledge proof, searchable-symmetric encryption, blockchain, and smart contracts. In terms of security, this scheme can guarantee the confidentiality, availability, tamper-proofability, and ciphertext retrievability of credit-investigation data, as well as the fairness and anonymity of identity authentication in the inquiry of credit-investigation data. In terms of efficiency, compared with similar identity-authentication schemes, in addition to the increase in the key generation time by about 225%, the proof key of this scheme was reduced by about 87.6%, and the time consumed for verification was reduced by about 82.9%. Compared with similar ciphertext-retrieval schemes, the index generation time of this scheme was reduced by about 66.7%, and the time for generating trapdoors and returning search results was reduced by about 98.7%. In the next work, we will focus on improving the efficiency of zero-knowledge proof.

## Figures and Tables

**Table 1:** Notation description.

Notation	Description	Notation	Description
*k*	Security parameters	$w_{i}$	Keyword
$D_{i}$	Credit data	δ(D)	Keyword set
$c_{w_{i}}$	Ciphertext of credit data	$K^{{}^{\prime }}$	Private-key ciphertext
π	Zero-knowledge proof	*I*	Index

**Table 2:** Comparison of system characteristics.

Scheme	Our Scheme	Scheme [9]	Scheme [10]
Confidentiality	√	√	√
Availability	√	√	√
Tamper-proof	√	√	√
Traceability	√	√	√
Ciphertext retrievability	√	×	×
Anonymous identity	√	×	×
Certification	√	×	√

**Table 3:** The time and constraints for the compilation, key generation, proof, and authentication of three types of input.

Input	Constraints	Compilation	Zero-Knowledge Key Generation	Generate Proof	Authentication
8	19	0.004 s	0.018 s	0.007 s	0.006 s
16	35	0.005 s	0.023 s	0.012 s	0.006 s
16-Sha256	27,479	1.878 s	16.435 s	2.051 s	0.006 s

**Table 4:** Size of proof key, verification key, and proof of three types of input.

Input	Proof Key	Verification Key	Proof
8	8.5 KB	2.5 KB	1.3 KB
16	15.1 KB	3.7 KB	1.8 KB
16-Sha256	6.4 MB	4.0 KB	1.9 KB

**Table 5:** Comparison between this scheme and scheme [18].

Scheme	Scheme [18]	This Scheme
Zero-knowledge key generation	7.3 s	16.435 s
Generate proof	1.65 s	2.051 s
Authentication	0.035 s	0.006 s
Proof key	51.7 MB	6.4 MB
Verification key	3.96 KB	4.0 KB

**Table 6:** The relative time-consuming of this scheme.

Operation	Compilation	Zero-Knowledge Key Generation	Generate Proof	Authentication	Build Index	Generate Trapdoors and Return Search Results	Total
Relatively time- consuming	1	8.754	1.092	0.003	1.064	0.008	11.921

## Data Availability

The data can be made available on request from the corresponding author.
